# Vitamin D, SARS-CoV-2 and Causal Associations in Transversal Studies: The Time-Series Analysis to Reveal Potential Confounders. Comment on Gaudio et al. Vitamin D Levels Are Reduced at the Time of Hospital Admission in Sicilian SARS-CoV-2-Positive Patients. *Int. J. Environ. Res. Public Health* 2021, *18*, 3491

**DOI:** 10.3390/ijerph18136793

**Published:** 2021-06-24

**Authors:** Cristiano Ialongo, Antonella Farina, Raffaella Labriola, Antonio Angeloni, Emanuela Anastasi

**Affiliations:** Department of Experimental Medicine, Policlinico Umberto I, ‘Sapienza’ University, 00185 Rome, Italy; antonella.farina@uniroma1.it (A.F.); raffaella.labriola@uniroma1.it (R.L.); antonio.angeloni@uniroma1.it (A.A.)

We read with great interest the paper by Gaudio and colleagues on vitamin D and on the state of patients with severe acute respiratory syndrome coronavirus 2 (SARS-CoV-2) at the time of admission [1]. The study involved a small cohort enrolled from March to July 2020, which was the time of the first wave of the pandemic. One point that we found interesting was that, despite the significant difference in median vitamin D levels between patients and controls (12.5 ng/mL and 20.5 ng/mL, respectively), the authors did not support the causal relationship between low levels of vitamin D and SARS-CoV-2. This is indeed a wise approach, especially since the authors recognized that the “cross-sectional and retrospective design may have limited our ability to establish the causality and temporality of associations”.

It is well known that the collection of data used in cross-sectional studies does not take into account their time-related structure, leading to the modeling of phenomena based on “differences” rather than “changes”. This implicitly assumes that the underlying phenomenon was stationary during the observation period—a gross oversimplification in many circumstances. Particularly with regard to the COVID-19 pandemic, we know that there has been a series of events that have alternated interventions and responses to the pandemic, introducing (or removing) factors that may have changed the nature and intensity of the aforementioned associations.

In light of the above, we decided to examine the data generated by our institution (Policlinico Umberto I, “Sapienza” University of Rome, Rome, Italy), which, since early during the outbreak of the pandemic, has operated as a “COVID-19 hospital”. In particular, we retrieved laboratory results of the serum vitamin D levels and combined IgG/IgM antibodies toward COVID-19 nucleocapsid protein (Ab-COV-N) of 247 subjects (161 males and 86 females, mean age 60.9 ± 17.5 years and 61.1 ± 17.8 years, respectively) measured at the time at which they were referred to our institution for symptoms and/or signs of SARS-CoV-2. All the subjects were then confirmed to have contracted SARS-CoV-2 by molecular analysis.

In order to preserve the structure of data and access the time-related information, we based our statistical analysis on segmented regression, which is a straightforward means of modeling time-series [2]. In particular, we applied a single changepoint structure in order to determine whether—and thus, eventually, when—either slope or intercept (or both) changed from baseline; in our model, baseline was represented by data collected during the period of national lockdown (from 9 March to 18 April 2020). The procedure sought first the changepoint for serum vitamin D by maximizing the adjusted R-square of the model, and then applied it to Ab-COV-N. Normality of data was determined using the Box–Cox power transformation (λ(AB-COV-N) = 0.2 and λ(vitamin D) = 0 or natural logarithm), and Statistica 10 for Windows (Stat-soft Inc., Tulsa, OK, USA) was used to carry out all the calculations.

The results of our analysis are displayed in panels “A” and “B” of Figure 1 and Table 1 for the parameters of the regression model with statistical analysis. The changepoint of serum vitamin D was located between 17 August and 26 August 2020. Under these conditions, there was a significant positive step-change for vitamin D and a negative step-change for Ab-COV-N. In particular, after back-transformation and interpolation were performed, the average vitamin D level was 9.6 ng/mL at baseline and 19.3 ng/mL by the changepoint. 

Two notable observations can be drawn from our findings: first, the changepoint fitted well with the step-change of both vitamin D level and Ab-COV-N, and second, the serum vitamin D level changed on average over the baseline from deficiency to almost sufficiency. As this occurred around the middle of August, we may speculate that the vitamin D level was influenced by environmental factors—for instance, sunlight exposure, which increased during May–June. However, the level of the antibody response for SARS-CoV-2 correlates with the time of the infection and not with the severity of the disease; therefore, this eliminates the possibility that the fall in Ab-COV-N was due to the increased immunomodulatory effect of the higher vitamin D level [3]. Thus, is there an explanation as to why, in the absence of any plausible mutual effect, we observed the same behavior for both markers?

If we consider the temporality of the phenomenon to obtain an insight into the causality of associations, it can be seen that, according to the national statistics on the COVID-19 pandemic, since mid-August, the number of subjects tested per day for SARS-CoV-2 suddenly changed [4]. In particular, by 18 August, this number had increased from around 25,000 to almost 50,000, and then steadily increased to almost 130,000 until mid-November. As panel “C” of Figure 1 shows, such a step-change almost coincides with the changepoint of both vitamin D level and Ab-COV-N of our data.

While any interpretation should be made with caution, a degree of speculation is unavoidable. Indeed, during the lockdown, the number of potential SARS-CoV-2 cases exceeded the actual capacity to test an adequate number of subjects. In these conditions, it is likely that the decision to refer a patient to hospital depended mainly (also due to the limited experience gained in those very first days) on the severity of the clinical picture or on how suggestive it was of SARS-CoV-2 [5]. Although unconfirmed, we can speculate that, due to the relationship of vitamin D with nutritional status and inflammatory modulation, this induced a positive selection bias towards those with low serum vitamin D. From mid-August onwards, however, the increased ability to perform specific tests made it easier to analyze anyone who might be suspected of carrying SARS-CoV-2. As a result, the selection bias disappeared regardless of its nature. Since the scarcity of tests certainly affected the timeliness with which the diagnosis of SARS-CoV-2 could be confirmed, Ab-COV-N in the baseline period essentially reflected the prevalence of IgG. Similarly, an earlier diagnosis as conceivable after mid-August led to shorter observation times from infection and therefore to a prevalence of IgM. Since IgG is more abundant than IgM, the change point appeared as a negative step-change in Ab-COV-N. 

A consequence of the hypothesis of the selection bias is that the levels of vitamin D in the patients (12.5 ng/mL) and controls (20.5 ng/mL) in the Gaudio study are, respectively, similar to those of our baseline (9.6 ng/mL) and post-changepoint cases (19.3 ng/mL) [1]. In particular, for the post-changepoint cases, which displayed unexpected results, their behavior suggests that the observed effect is only that of the factors that regulate vitamin D status in the general population (such as oral supplementation, age, comorbidities, diet, etc.), a random sample of which is represented by the controls in the Gaudio study.

The selection bias could explain why there appears to be so many studies conducted on Italian cohorts before mid-August 2020 (including Gaudio’s) that disagree on the role of vitamin D status in SARS-CoV-2 [1,6,7,8,9,10,11]. In fact, the impact of the COVID-19 outbreak and related testing capacity varied across Italy, and therefore the selection bias towards low vitamin D status may have been different in different cohorts. Unfortunately, we have not been able to access the local data for the number of subjects tested each day, and therefore, especially in this case, our conclusions are only suggestions.

Thus, the work of Gaudio and his collaborators is important above all because it highlights two critical issues concerning the nature of the relationship between vitamin D and SARS-CoV-2:

(1) It is objectively easier to draw biased conclusions due to an unrecognized biased selection of cases, rather than establishing a causal relationship by studying a single biological marker or even controlling all the possible (confounding) factors.
(2) The cross-sectional design (through the comparison of means or medians) is always inadequate for studies that deal with a phenomenon that is subject to a complex temporal dynamics with multiple changes.

On this basis, the ability to establish correctly the relationship between “causality and temporality of associations” is an essential factor to support the possible lack of association between susceptibility to COVID-19 and serum vitamin D.

## Figures and Tables

**Figure 1:**
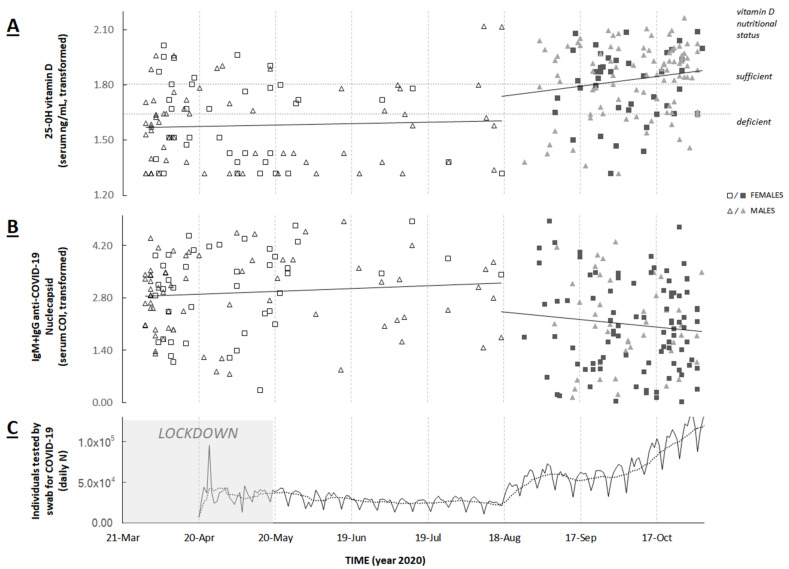
Summary of serum 25-OH vitamin D (**A**), IgM+IgG anti-COVID-19 nucleocapsid protein (**B**) and number of individuals tested per day by swab for COVID-19 (**C**); (panels **A**,**B**): solid lines represent the trend and level of the segment regression (statistical analysis is detailed in Table 1); the dotted horizontal lines in panel B represent the transformed reference values of vitamin D status corresponding to sufficient (=20 ng/mL) and deficient (=12 ng/mL) condition; (panel **C**): the gray shaded area represents the period of lockdown; the grey dotted line is the 7-day mobile average overlapped with the daily number of tested subjects (N).

**Table 1:** Segmented regression analysis with single changepoint (transformed values).

Parameters	Serum 25-OH Vitamin D				IgM+IgG Anti-COVID-19 Nuclecapsid Protein N (Ab-COV-N)			
	Coefficient (*B*)	std.Error	*t*-Statistic	*p*-Value	Coefficient (*B*)	std.Error	*t*-Statistic	*p*-Value
baseline level	1.572	0.026	60.193	<0.001	2.826	0.151	18.751	<0.001
baseline slope	9.25 × 10^−5^	0.000	0.186	0.852	0.004	0.003	1.224	0.222
step-change *	0.149	0.074	2.011	0.045	−0.862	0.427	−2.02	0.044
trend-change *	0.002	0.001	1.634	0.103	−0.010	0.006	−1.726	0.086

* changepoint: 18 August 2020.

## References

[1] Gaudio A., Murabito A.R., Agodi A., Montineri A., Castellino P., D.O.CoV Research (2021). Vitamin D Levels Are Reduced at the Time of Hospital Admission in Sicilian SARS-CoV-2-Positive Patients. Int. J. Environ. Res. Public Health.

[2] Wagner A.K., Soumerai S.B., Zhang F., Ross-Degnan D. (2002). Segmented regression analysis of interrupted time series studies in medication use research. J. Clin. Pharm. Ther..

[3] Zhou W., Xu X., Chang Z., Wang H., Zhong X., Tong X., Liu T., Li Y. (2021). The dynamic changes of serum IgM and IgG against SARS-CoV-2 in patients with COVID-19. J. Med. Virol..

[4] Lab24 Coronavirus in Italy, Dates and Maps. https://lab24.ilsole24ore.com/coronavirus.

[5] Farina A., Labriola R., Ialongo C., Suppa M., Viggiani V., Lucarelli M., Anastasi E., Angeloni A. (2021). Transient plasma cell dyscrasia in COVID-19 patients linked to IL-6 triggering. MicrobesInfect.

[6] Ferrari D., Locatelli M. (2021). No significant association between vitamin D and COVID-19. A retrospective study from a northern Italian hospital. Int. J. Vitam. Nutr. Res..

[7] Cereda E., Bogliolo L., Lobascio F., Barichella M., Zecchinelli A.L., Pezzoli G., Caccialanza R. (2021). Vitamin D supplementation and outcomes in coronavirus disease 2019 (COVID-19) patients from the outbreak area of Lombardy, Italy. Nutrition.

[8] Barassi A., Pezzilli R., Mondoni M., Rinaldo R.F., DavÌ M., Cozzolino M., Melzi D’Eril G.V., Centanni S. Vitamin D in severe acute respiratory syndrome coronavirus 2 (SARS-CoV-2) patients with non-invasive ventilation support. Panminerva Med..

[9] Giannini S., Passeri G., Tripepi G., Sella S., Fusaro M., Arcidiacono G., Torres M.O., Michielin A., Prandini T., Baffa V. (2021). Effectiveness of In-Hospital Cholecalciferol Use on Clinical Outcomes in Comorbid COVID-19 Patients: A Hypothesis-Generating Study. Nutrients.

[10] Carpagnano G.E., Di Lecce V., Quaranta V.N., Zito A., Buonamico E., Capozza E., Palumbo A., Di Gioia G., Valerio V.N., Resta O. (2021). Vitamin D deficiency as a predictor of poor prognosis in patients with acute respiratory failure due to COVID-19. J. Endocrinol. Investig..

[11] Infante M., Buoso A., Pieri M., Lupisella S., Nuccetelli M., Bernardini S., Fabbri A., Iannetta M., Andreoni M., Colizzi V. Low Vitamin D Status at Admission as a Risk Factor for Poor Survival in Hospitalized Patients With COVID-19: An Italian Retrospective Study. J. Am. Coll. Nutr..

